# Changes in blood biomarkers correlate with changes in cardiac size and function in patients with tetralogy of Fallot

**DOI:** 10.1016/j.ijcchd.2024.100522

**Published:** 2024-06-17

**Authors:** Wouter J. van Genuchten, Eva van den Bosch, Saskia E. Luijnenburg, Vivian P. Kamphuis, Jolien W. Roos-Hesselink, Beatrijs Bartelds, Arno A.W. Roest, Johannes M.P.J. Breur, Nico A. Blom, Eric Boersma, Laurens P. Koopman, Willem A. Helbing

**Affiliations:** a Erasmus University Medical Center, Department of Pediatrics, Division of Pediatric Cardiology, Rotterdam, the Netherlands; b Erasmus University Medical Center, Department of Radiology, Rotterdam, the Netherlands; c Netherlands Heart Institute, Utrecht, the Netherlands; d Leiden University Medical Center, Department of Pediatrics, Division of Pediatric Cardiology, Leiden, the Netherlands; e Erasmus University Medical Center, Department of Cardiology, Rotterdam, the Netherlands; f University Medical Center Utrecht, Department of Pediatric Cardiology, Utrecht, the Netherlands; g Academic Medical Center, Department of Pediatrics, Division of Pediatric Cardiology, Amsterdam, the Netherlands; h Erasmus University Medical Center, Department of Pediatrics, Rotterdam, the Netherlands

## Abstract

**Introduction:**

Patients after surgical correction of Tetralogy of Fallot (ToF) often show adverse cardiac remodeling. To better understand the underlying biological processes, we studied the relation between changes in blood biomarkers and changes in biventricular size and function as assessed by cardiac magnetic resonance imaging (CMR).

**Methods:**

This study included 50 ToF patients, who underwent blood biomarker and CMR analysis at least twice between 2002 and 2018.34 (68 %) of these patients were male. Patients had an average age of 16.1 at first visit. Biomarkers were chosen based on earlier research by our group and included: NT-proBNP, ST2, GDF-15, DLK-1, IGFBP-1/7, and FABP-4. Pearson correlations coefficients (r_pearson_) were determined to quantify the relationship between changes in biomarkers and CMR measurements.

**Results:**

For changes in parameters of right ventricular (RV) size significant correlations were observed with changes in NT-proBNP, ST-2, GDF-15, IGFBP7 and FABP-4 (r_pearson_ between 0.28 and 0.51). Correlations with NT-proBNP were driven by changes in RV size induced by pulmonary valve replacement (n = 9). For LV serial size changes, significant correlations were noted with changes in NT-pro-BNP, ST-2, GDF-15 and FABP-4 (r_pearson_ between 0.32 and 0.52).

**Conclusion:**

In clinically stable ToF patients changes in right and left ventricular size and function correlated with alterations in blood biomarkers of inflammation and immune response to stress.

## Highlights

• Our study explores relationships in changes in blood biomarkers and changes in biventricular size/function in ToF patients.
• Correlations were found between changes in ventricular size and changes in NT-proBNP, ST-2, GDF-15, IGFBP7 and FABP-4.
• The correlations with NT-proBNP were driven by changes in right ventricular size induced by pulmonary valve replacement.

## 1 Introduction

Improvements in antenatal screening, diagnostic and surgical techniques and supportive care have resulted in increased survival of patients with congenital heart disease (ConHD) [1,2]. However, long-term complications such as reoperations, rhythm disorders, heart failure and sudden cardiac death are common after corrective surgery [[1], [2], [3]]. The underlying pathophysiology leading to these complications is incompletely understood. The American Heart Association deemed a better understanding of the underlying mechanisms of right ventricular (RV) remodeling one of the high-impact questions in ConHD [4].

In patients with ischemic heart disease, blood biomarkers play a major role in understanding the pathophysiology and predicting long-term outcomes and have become an integral part of diagnostic work-up [5]. Recently, various biomarkers have been explored in ConHD, including markers associated with cardiac remodeling, myocardial stress, fibrosis, inflammation, and vascularization pathways [[6], [7], [8], [9]]. However, due to the cross-sectional design of these studies, the relationships between changes in cardiac size and function, outcomes and concurrent changes in blood biomarkers are not well understood. Tetralogy of Fallot (ToF) is associated with one of the highest incidences of heart failure of all types of ConHD [3]. Heart failure in ToF patients relates primarily to abnormal RV loading conditions, most commonly from volume overload related to chronic pulmonary insufficiency, a common sequela of primary surgical correction [2,3,10]. The gold standard for quantifying RV cardiac size and function in patients with ToF is cardiac magnetic resonance imaging (CMR) [10].

The aim of this study was to explore the relationship between changes in blood biomarkers to changes in biventricular size over time, to better understand the underlying pathofysiology of cardiac remodeling in ToF patients.

## 2 Methods

We included patients with ToF who in whom blood biomarkers and CMR were assessed at two time-points during serial follow-up after complete surgical correction. Data was acquired in the setting of several cross-sectional and prospective studies at one of five contributing tertiary referral centers between 2002 and 2018 [[11], [12], [13], [14]]. The institutional review board of the Erasmus Medical Center approved the studies (METC 2006–310, 2009–033, 2009–134, 2014–326). All participants, and if necessary their parents (age under 16), gave written informed consent before inclusion in these studies. The patients were subsequently followed in regular clinical follow-up in the participating centers.

Blood samples were drawn from a peripheral vein and collected in EDTA tubes at the time of CMR. Samples were stored at −80 °C. The frozen samples were shipped to Olink Proteomics AB (Uppsala, Sweden) for analysis with the Olink® Target 96 Cardiovascular panel III. Using proximity extension assay (PEA) technology, the levels of biomarkers were measured. This PEA technique has been described extensively [22]. Laboratory staff was blinded to the patients’ clinical and study data. Biomarker values are reported as normalized protein expression (NPX) units on a Log 2 scale. For this current study, we examined N-terminal pro b-type natriuretic peptide (NT-proBNP), soluble interleukin 1 receptor-like 1 (ST2), growth differentiation factor 15 (GDF-15), delta like non-canonical Notch ligand 1 (DLK-1), von Willebrand factor (VWF), insulin-like growth factor-binding protein 1 and 7 (IGFBP-1/7), and fatty acid binding protein 4 (FABP-4). We have shown these markers to relate to cardiac function and long term outcome in earlier studies [9,15].

CMR was performed on the same day as blood sampling according to a standardized protocol in all contributing centers. This protocol has been described previously [11,16,17]. In brief, a set of short-axis images, acquired using balanced steady-state free precession (bSSFP) cine imaging, was taken from base to apex. Slices were planned on the four-chamber image, parallel to the atrioventricular valve plane of the left ventricle in end-diastole. The typical imaging parameters included a repetition time of 3.4 ms, an echo time of 1.5 ms, a flip angle of 45°, a slice thickness of 7–10 mm, an inter-slice gap of 0–1 mm, a field of view of 380 × 380 mm, a phase field of view of 0.7. All images were acquired during a breath-hold in end-expiration. MASS and FLOW (Medis Medical Imaging Systems, Leiden, and The Netherlands) were used to perform the analysis. All CMRs were analysed by one of the authors (E.v.d.B.) under the supervision of W.H. Papillary muscles and trabeculae were included in the ventricular mass.

Continuous variables are summarized as mean value ± SD, or as median value (25th–75th percentile). For categorical variables, both absolute numbers and percentages are reported. Measured biomarker values and CMR parameters were used to calculate the annual change. We then studied the correlation between these changes, reporting Pearson's correlation coefficient (r_Pearson_). We first performed these analyses on the entire group of patients. We then selected the significant correlations and reran the analyses on the patients who did not undergo a pulmonary valve replacement (PVR) as these patients did not have a complete change in physiology between measurements. Analyses were performed using R (version 4.2.1) [18]. We considered P < 0.05 as statistically significant.

## 3 Results

A total of 34 and 16 male and female ToF patients were available for analysis, with a median age of 16.1 years at the baseline study visit (Table 1). The median age at ToF repair was 11 months, and 31 (62 %) were operated using a transannular patch. Staged repair was performed in 7 (14 %) patients. Three patients (6 %) had 22q11 deletion syndrome.

**Table 1:** Demographic and CMR parameters at visits.

	Visit 1		Visit 2	
	Mean/Median/N	SD/25th-75th Percentile/%	Mean/Median/N	SD/25th-75th Percentile/%
AGE AT VISIT (YEARS)	16.1	12.4–21.1	24.0	19.4–30.6
TIME AFTER TOF REPAIR (YEARS)	15.1	11.8–20.0	23.1	18.9–29.7
TIME BETWEEN VISIT 1 AND 2 (YEARS)	7.0	5.2–8.3		
CMRI				
RV EDV (ML/M^2^)	128	36	122	37
RV ESV (ML/M^2^)	64	24	61	23
RV EF (%)	52	6	50	5
RV SV (ML/M^2^)	66	16	61	16
RV MASS (G)	24	8	23	6
RV MASS/EDV RATIO (G/ML/M^2^)	0.20	0.07	0.18	0.04
PR (%)	28	7–38	20	3–36
LV EDV (ML/M^2^)	83	11	86	13
LV ESV (ML/M^2^)	33	7	37	8
LV EF (%)	61	6	58	6
LV SV (ML/M^2^)	51	7	49	9
LV MASS (G)	53	8	52	7
LV MASS/EDV RATIO (G/ML/M^2^)	0.66	0.10	0.62	0.09

Abbreviations:CMRI:cardiac magnetic resonance imagingEDV:end-diastolic volumeEF:ejection fractionESV:end-systolic volumeLV:left ventricularPR:pulmonary regurgitationRV:right ventricularSV:stroke volume.

At baseline visit, mean CMR-derived right ventricular end-diastolic volume (RVEDV) was 128 ml/m^2^ (standard deviation [SD] 36), mean right ventricular ejection fraction (RVEF) 52 % (SD 6), pulmonary regurgitation % (PR) median 28 (25th-75th percentile: 7–38). For more details and left ventricle data see Table 1.

The follow-up measurements took place after a median of 7.0 years (25th-75th percentile: 5.2–8.3). Then, mean RVEDV was 122 ml/m^2^ (SD 37), mean RVEF 50 % (SD 5), and median PR 20 % (25th-75th percentile: 3–36). In the nine (18 %) patients who underwent PVR between the two visits, mean RVEDV decreased from 160 ml/m^2^ to 114 ml/m^2^. Mean RVEDV remained largely unchanged in patients without PVR (121 ml/m^2^ versus 124 ml/^2^). Details on CMR measurements are reported in Supplemental Tables 1 and 2, whereas Supplementary Tables 4 and 5 show the measured biomarkers.

**Fig. 1:**
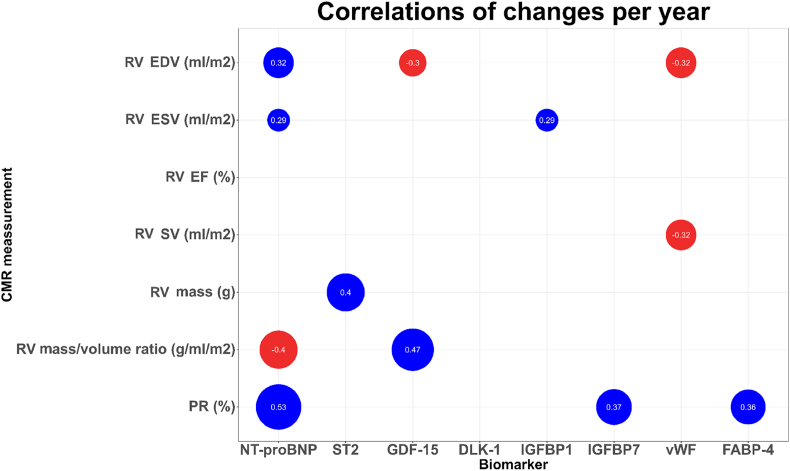
Correlation coefficients for statistically significant relations between changes in blood biomarkers and magnetic resonance imaging assessment of right ventricular size and function and pulmonary regurgitation (n = 50). Positive correlations are displayed blue, negative correlations red Abbreviations: EDV: end-diastolic volume EF: ejection fraction ESV: end-systolic volume PR: pulmonary regurgitation RV: right ventricular SV: stroke volume.

Fig. 1 displays correlations between the estimated annual changes in ventricular size (CMR-based volume and mass) and function (ejection fraction [EF]) versus annual changes in blood biomarkers. Our findings demonstrate that changes in RVEDV are positively correlated with changes in NT-proBNP (r = 0.32, p = 0.03) and negatively with GDF-15 (r = 0.3, p = 0.04) and VWF (r = −0.32, p = 0.03). RVESV changes correlated to NT-proBNP (r = 0.29, p = 0.049) and IGFBP1 (r = 0.29, p = 0.049). RVSV was inversely correlated to changes in VWF(r = −0.32, p = 0.03) whereas RV mass changes correlated to changes in ST2 (r = 0.40, p = 0.02). Changes in right ventricular mass to volume ratio correlated to both NT-proBNP (r = −0.40, p 0.02) and GDF-15 (r = 0.47, p 0.008). Alterations in PR correlate to NT-proBNP (r = 0.53, p = 0.0007), IGFBP-7 (r = 0.37, p = 0.02) as well as FABP-4 (r = 0.36, p = 0.03).

In the patients who did not undergo PVR between both measurements, correlations between changes in RVEDV/RVESV and NT-ProBNP were not significant, nor was the correlation between ST2 and RVmass (Supplemental Table 3). Notably, the correlation between GDF-15 and RVEDV increased from −0.30 to −0.55 in this group, and the correlation between IGFBP7 and PR increased from 0.37 to 0.77. All other correlations remained relatively similar.

**Fig. 2:**
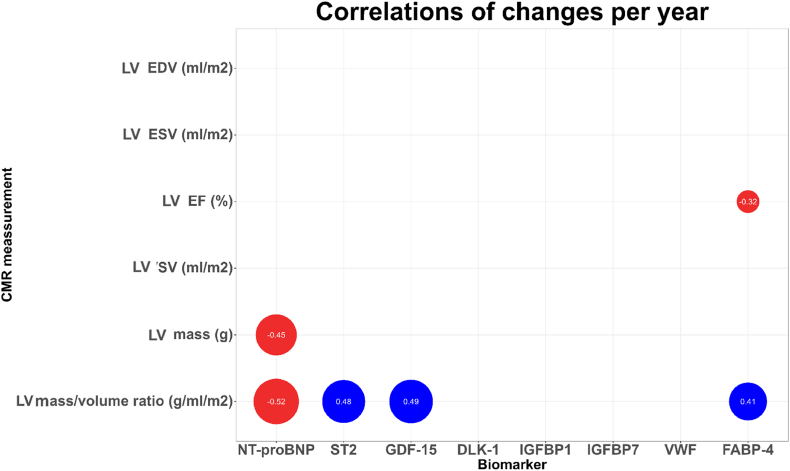
Correlation coefficients for statistically significant relations between changes in blood biomarkers and magnetic resonance imaging assessment of left ventricular size and function and pulmonary regurgitation (n = 50). Positive correlations are displayed blue, negative correlations red Abbreviations: EDV: end-diastolic volume EF: ejection fraction ESV: end-systolic volume PR: pulmonary regurgitation LV: left ventricular SV: stroke volume.

Fig. 2 shows correlations between changes in blood biomarkers and changes in left ventricular CMR measurements. Increases in LV mass (LVM) were correlated to decreases in NT-proBNP (r = −0.45, p = 0.01). Increases in left ventricular mass-to-volume ratio (LVMVR) were positively correlated to increases in: ST-2 (r = 0.48, p = 0.006), GDF-15 (r = 0.49, p = 0.004) and FABP4 (r = 0.41, p = 0.02) and negatively correlated to NT-proBNP. Lasty, we found a negative association between changes in LVEF and changes in FABP-4 (r = 0.32, p = 0.03).

## 4 Discussion

To the best of our knowledge our findings are the first to demonstrate correlations between serial changes in right ventricular size and function and alterations in blood biomarkers in patients with Tetralogy of Fallot. For the individual patients, increases in right ventricular size and mass correlated with an increase NT-proBNP, ST2 and IGFBP-7 and a decrease in GDF-15. An increase in PR was associated with iincreasing levels of IGFBP-7 and FABP-4. Correlations between RV size, PR and function and IGFBP-7, GDF-15 and FABP-4 remained significant if we excluded the PVR group, whereas in this non-PVR group correlations were not significant anymore for NT-proBNP and ST2.

Subsequently we will discuss the biological role and known relationships to outcome or cardiac structure for each biomarker we tested.

### 4.1 NT-proBNP

NT-proBNP is secreted in response to increased myocardial stretch, ventricular volume, and pressure overload [19]. It serves as a well-known biomarker in acquired heart failure and adult ConHD patients, where elevated levels are linked to mortality and adverse events [19]. In an earlier study by our group, increased NT-proBNP levels were related to adverse events in patients with corrected ToF [9]. NT-proBNP increases are also correlated to adverse events in cohorts consisting of patients with various types of conHD [2,19,20]. Based on these results and observations in our current cohort, NT-proBNP is a marker of the underlying right ventricular adverse remodeling caused by the PR. In our study decreases in LVM and LVMVR were correlated with increases in NT-proBNP. In the left ventricle higher NT-ProBNP is generally related to increased LVM [21,22]. In patients with ToF higher RVEDV and PR have been shown to correlate with lower LV function, lower LVmass and higher LV volumes [[23], [24], [25]] possibly mediated through ventriculo-ventricular interaction and electrophysiological abnormalities [26]. This illustrates the complex interaction between RV and LV size and function in ToF with PR [27].

### 4.2 ST2

ST2 is a member of the interleukin-1 receptor family and exists in both soluble and transmembrane forms [6,28]. It is upregulated in response to myocardial stress and serves as a marker for inflammation, remodeling, fibrosis, and apoptosis in the myocardium [6,28,29]. In adult cohorts with complex ConHD, elevated soluble ST2 levels have been observed, predicting all-cause mortality and major adverse events during follow-up when corrected for NT-proBNP level [6]. A negative correlation between soluble ST2 levels and left ventricular EF was observed in a pediatric ConHD cohort that included various defects [30]. A previous study by our groups showed increased adverse events in patients with increased ST2 in a population of patients with univentricular hearts [15]. In the current cohort, increases in ST2 were correlated with increases in RV mass, which is a known predictor of death and sustained ventricular tachycardia in patients with ToF [31]. Our findings are in line with prior research indicating a similar association between ST2 and increased LVM and LMVR in patients with hypertrophic cardiomyopathy [32].

### 4.3 GDF-15

GDF-15 is a cytokine belonging to the transforming growth-factor-β family and is associated with acquired heart disease [33]. Several studies have shown that elevated levels of GDF-15 are associated with all-cause mortality in patients with heart failure caused by acquired heart disease, independent of known prognostic variables [34]. The evidence for a role of GDF-15 in patients with ConHD is mixed. Some studies showed an association with mortality, heart failure and adverse events [35,36]. Other studies showed no associations with outcomes [9]. In our cohort increases in GDF-15 were associated with decreases in RVEDV which is generally regarded positive in patients with ToF. Therefore, the results of our findings should be repeated in other cohorts for a more comprehensive understanding of adverse remodeling.

### 4.4 IGFBP-1/7

IGF binding proteins (IGFBPs) are a family of proteins that regulate and modulate IGF activity, indirectly affecting growth hormone [37]. IGBPs are also known to influence the immune response in heart failure cohorts [38]. Among them, IGFBP-7 is highly expressed in endothelial cells and has been linked to collagen deposition [39]. IGFBP-1 is seen as a marker of myocardial damage and related to heart failure [40]. Notably, IGFBP-7 has also been associated with post-infarction myocardial repair. In acquired heart failure patients, IGFBP-7 has been identified as a potential biomarker for adverse outcomes and is linked with diastolic dysfunction and lower VO2 max [39,41]. However, the role of IGFBPs in cardiac function or prognosis in patients with ConHD is largely unexplored. Nevertheless, IGFBPs have been linked to general growth, failure to thrive, nutritional status, and subclinical kidney injury in this patient population [42,43]. In an earlier study, our group found that IGFBP-7 was related to adverse events in patients with ToF [9]. This corresponds with the correlation we found in this study between increases in PR and IGFBP-7.

### 4.5 FABP-4

As shown in previous research, FABP-4 is highly expressed in adipocytes, and its increased levels have been associated with adiposity, hypertension and diabetes [41,44]. Additionally, FABP-4 displays some expression in macrophages, and enhances foam cell formation and triggers an inflammatory response [44]. FABP-4 is correlated to atherosclerosis and heart failure, although less is known in patients with ConHD [45].

Previous studies have linked elevated FABP-4 levels to LV hypertrophy, heart failure, and both systolic and diastolic dysfunction [41,44]. Moreover, an observed increased expression of FABP-4 RNA in the right ventricle of ToF patients, compared to VSD patients, implies a role of FABP-4 in adverse RV remodeling in patients with ToF [46].

Previous studies by our group found a negative association between FABP-4 levels and peak VO2 in both Fontan and Fallot cohorts [9,15]. Our current study found a positive association between increases in FABP-4 levels and increases of PR over time. This suggests that FABP-4 plays a role in the underlying pathophysiological pathways, leading to remodeling which may affect exercise performance in ToF patients. FABP-4 has been correlated to increased LMVR, however the relationship between an increase in FABP-4 and a decrease in LVEF, as noted in our patients, has not been described in literature.

### 4.6 Clinical and research implications

Serial assessment of these biomarkers may lead to better monitoring of disease progression and track the effect of therapeutic interventions such as PVR. Especially NT-proBNP correlated strongly to the decrease in RVEDV and RVESV seen after a PVR. Furthermore, our data highlights the potential utility of relatively novel biomarkers GDF-15, ST2, IGFBP-1, IGFBP-7 and FABP-4 as additional biomarkers that could contribute to a more comprehensive understanding of the pathophysiology underlying ConHD. All these biomarkers are involved in the response to (cardiac) stress such as inflammation and/or immune, responses or metabolic changes associated with remodeling [6,28,29,38,45]. The relationship between inflammatory cytokines and functional status of patients with ConHD has first been described by the group of Imperial College London [47]. These and our findings might provide potential new therapeutic agents, which are required since the evidence for beneficial effects of “classical” heart failure drugs in congenital heart failure is limited [4]. In coronary artery disease immunomodulators such as colchicine have been suggested to be effective in improving mortality [48]. However the evidence is not conclusive in patients with heart failure [49,50].

Some of these biomarkers, e.g. FABP-4 and IGFBP's, might also be expressed outside of the heart. This could be a secondary effect related to suboptimal perfusion of other organs than the heart, which has been well described in Fontan patients and heart failure in acquired heart disease [51,52].

Before these findings can be translated into clinical practice further studies should focus on confirming our findings in larger (multi-center) cohorts, unravelling the relationship between biological process, changes in blood biomarkers and right ventricular remodeling.

### 4.7 Limitations

There are several limitations to this study. The sample size was relatively small, and this may have limited the power to detect smaller yet relevant correlations. Furthermore, the sample size prervented separate analysis of the PVR group. Lastly the sample size was too small to relate changes in biomarkers to clinical outcome data. However, this is the largest study investigating serial measurement of blood biomarkers in patients with ToF. The sample size of this study warrants that results are confirmed in larger cohorts. We were limited to the biomarkers included in Olink biomarker panel. We might have explored biomarkers pertaining to the renin-angiotensin-aldosterone system, or more general markers of renal function like creatinine and plasma sodium concentration [53,54].

Furthermore, we did not have access to myocardial tissue, limiting our analysis to measurable blood biomarkers, which are derivatives of underlying processes that could be measured more directly by e.g. myocardial proteomics and/or RNA sequencing. Whilst difficult to obtain in all stages of disease, analysis of myocardial tissue might provide novel insights of underlying remodeling in ToF patients [55].

## 5 Conclusion

In conclusion, our study demonstrates significant correlations between serial changes in blood biomarkers, such as NT-proBNP, GDF-15, ST2, IGFBP-1, IGFBP-7, and FABP-4, and alterations over time in right ventricular size and function as assessed by CMR imaging in patients with ToF. These findings suggest that these blood biomarkers might play a role in future monitoring of disease progression and improving prognostication in this patient population. Furthermore, our data highlight the role of inflammation and immune responses in cardiac remodeling in ToF, pointing towards potential directions for the development of targeted therapies and personalized management strategies for these patients.

## Funding

E. van den Bosch, V.P. Kamphuis, J.W. Roos-Hesselink and S. E. Luijnenburg were supported by research grants from the Dutch 10.13039/100002129Heart Foundation (grant 2013T091, grant 2013T093, grant 2008B026 and grant 2006B095).

W.J. van Genuchten was supported by the Netherlands Cardiovascular Research initiative: an initiative with support of the Dutch 10.13039/100002129Heart Foundation and Hartekind (grant to WA Helbing as part of a consortium; CVON219-002 OUTREACH) B. Bartelds is supported by a grant from the Dutch 10.13039/100002129Heart Foundation, Clinical Established Investigator grant 03-001-2021-T105.

## Disclaimer

Jolien W. Roos-Hesselink is an Editorial Board Member of the International Journal of Cardiology Congenital Heart Disease and played no role in the Journal's evaluation of the manuscript.

## CRediT authorship contribution statement

**Wouter J. van Genuchten:** Writing – original draft, Methodology, Investigation, Formal analysis, Data curation. **Eva van den Bosch:** Writing – review & editing, Investigation, Data curation, Conceptualization. **Saskia E. Luijnenburg:** Writing – review & editing, Investigation, Data curation. **Vivian P. Kamphuis:** Writing – review & editing, Investigation, Data curation. **Jolien W. Roos-Hesselink:** Writing – review & editing, Resources, Investigation. **Beatrijs Bartelds:** Writing – review & editing, Investigation. **Arno A.W. Roest:** Writing – review & editing, Investigation. **Johannes M.P.J. Breur:** Writing – review & editing, Investigation. **Nico A. Blom:** Writing – review & editing, Investigation. **Eric Boersma:** Writing – review & editing, Methodology. **Laurens P. Koopman:** Writing – review & editing, Software, Investigation. **Willem A. Helbing:** Writing – review & editing, Writing – original draft, Supervision, Resources, Project administration, Investigation, Funding acquisition, Conceptualization.

## Declaration of competing interest

The authors declare the following financial interests/personal relationships which may be considered as potential competing interests. WA Helbing reports financial support was provided by Netherlands 10.13039/100002129Heart Foundation. If there are other authors, they declare that they have no known competing financial interests or personal relationships that could have appeared to influence the work reported in this paper.
