# Supplementary material for: Changes in blood biomarkers correlate with changes in cardiac size and function in patients with tetralogy of Fallot
Source: Int J Cardiol Congenit Heart Dis. 2024 Jun 17;17:100522. doi: 10.1016/j.ijcchd.2024.100522 (PMC11657620; doi:10.1016/j.ijcchd.2024.100522)
Supplement: Multimedia component 1 [file mmc1.docx]

**Supplemental table 1: PVR group**

|  |  |  |  |  |
| --- | --- | --- | --- | --- |
| Parameter | SD / 25th-75th Percentile / % |  |  |  |
|  | N = 9 |  |  |  |
| Male (n, (%)) | 6 (66 %) |  |  |  |
| Age at ToF repair (months) | 11.5 (7.0-17.7) |  |  |  |
| Age at PVR (years) | 18.6 (4.8) |  |  |  |
| Transannular patch (n (%)) | 8 (88%) |  |  |  |
| Staged repair (n (%)) | 1 (11%) |  |  |  |
| 22q11 abnormality (n (%)) | 0 (0%) |  |  |  |
|  |  |  |  |  |
|  |  |  |  |  |
|  | **Visit 1** | | **Visit 2** | |
|  | Mean / Median / N | SD / 25th-75th Percentile / % | Mean / Median / N | SD / 25th-75th Percentile / % |
| RV EDV (ml/m^2^) | 160 | 43 | 114 | 32 |
| RV ESV (ml/m^2^) | 84 | 28 | 58 | 19 |
| RV EF (%) | 48 | 4 | 50 | 3 |
| RV SV (ml/m^2^) | 76 | 15 | 56 | 14 |
| RV mass (g) | 25 | 5 | 23 | 4 |
| RV mass / EDV ratio (g/ml/m^2^) | 0.16 | 0.04 | 0.21 | 0.05 |
| PR (%) | 40 | 33 - 49 | 3 | 0 - 21 |
|  |  |  |  |  |
| LV EDV (ml/m^2^) | 88 | 11 | 84 | 8 |
| LV ESV (ml/m^2^) | 36 | 5 | 36 | 4 |
| LV EF (%) | 58 | 3 | 57 | 4 |
| LV SV (ml/m^2^) | 51 | 7 | 49 | 7 |
| LV mass (g) | 52 | 11 | 50 | 7 |
| LV mass / EDV ratio (g/ml/m^2^) | 0.59 | 0.12 | 0.60 | 0.10 |
|  |  |  |  |  |

EDV: end-diastolic volume

EF: ejection fraction

ESV: end-systolic volume

PR: pulmonary regurgitation

RV: right ventricular

SV: stroke volume

PVR: pulmonary valve replacement

ToF: tetralogy of Fallot

| Supplemental table 2: non-PVR group | | | |  |
| --- | --- | --- | --- | --- |
|  |  |  |  |  |
| Parameter | SD / 25th-75th Percentile / % |  |  |  |
|  | N = 41 |  |  |  |
| Male (n (%)) | 28 (68 %) |  |  |  |
| Age at ToF repair (months) | 10.0 (5.3-18.0) |  |  |  |
| Transannular patch (n (%)) | 23 (66 %) |  |  |  |
| Staged repair (n (%)) | 7 (17%) |  |  |  |
| 22q11 Abnormality (n (%)) | 3 (7%) |  |  |  |
|  |  |  |  |  |
|  |  |  |  |  |
|  | **Visit 1** | | **Visit 2** | |
|  | Mean / Median / N | SD / 25th-75th Percentile / % | Mean / Median / N | SD / 25th-75th Percentile / % |
| RV EDV (ml/m2) | 121 | 31 | 124 | 38 |
| RV ESV (ml/m2) | 59 | 20 | 62 | 24 |
| RV EF (%) | 53 | 7 | 51 | 6 |
| RV SV (ml/m2) | 63 | 16 | 62 | 16 |
| RV mass (g) | 24 | 9 | 24 | 7 |
| RV mass / EDV ratio (g/ml/m2) | 0.20 | 0.05 | 0.18 | 0.03 |
| PR (%) | 25 | 4-32 | 28 | 4-40 |
|  |  |  |  |  |
| LV EDV (ml/m2) | 88 | 11 | 86 | 13 |
| LV ESV (ml/m2) | 32 | 7 | 37 | 8 |
| LV EF (%) | 61 | 6 | 58 | 6 |
| LV SV (ml/m2) | 50 | 7 | 50 | 9 |
| LV mass (g) | 54 | 9 | 52 | 7 |
| LV mass / EDV ratio (g/ml/m2) | 0.63 | 0.08 | 0.67 | 0.10 |
|  |  |  |  |  |

EDV: end-diastolic volume

EF: ejection fraction

ESV: end-systolic volume

PR: pulmonary regurgitation

RV: right ventricular

SV: stroke volume

PVR: pulmonary valve replacement

ToF: tetralogy of Fallot

**Supplemental table 3: correlations between changes in blood biomarkers and right sided CMR measurements**

| All patients (n=50) | | | | | | | | | |
| --- | --- | --- | --- | --- | --- | --- | --- | --- | --- |
|  | NT-proBNP | ST2 | | GDF-15 | DLK-1 | IGFBP1 | IGFBP7 | vWF | FABP-4 |
| RV EDV (ml/m^2^) | **0.32 (0.028)** | | -0.16 (0.276) | **-0.30 (0.045)** | -0.24 (0.115) | 0.09 (0.533) | 0.28 (0.057) | **-0.32(0.030)** | -0.12 (0.422) |
| RV ESV (ml/m^2^) | **0.29 (0.050)** | | -0.08 (0.597) | -0.27 (0.068) | -0.26 (0.086) | -0.02 (0.912) | **0.29 (0.049)** | -0.13 (0.376) | -0.06 (0.714) |
| RV EF (%) | 0.28 (0.060) | | -0.19 (0.218) | -0.26 (0.082) | -0.07 (0.627) | 0.15 (0.311) | 0.23 (0.128) | **-0.32 (0.028)** | -0.12 (0.440) |
| RV SV (ml/m^2^) | -0.17 (0.249) | | 0.01 (0.946) | 0.03 (0.847) | 0.28 (0.055) | 0.13 (0.395) | -0.1 (0.523) | -0.16 (0.301) | 0 (0.991) |
| RV mass (g) | -0.13 (0.499) | | **0.40 (0.024)** | 0.35 (0.051) | 0.14 (0.468) | -0.23 (0.218) | -0.23 (0.218) | -0.05 (0.790) | 0.35 (0.051) |
| RV mass / EDV ratio (g/ml/m^2^) | **-0.38 (0.034)** | | 0.34 (0.062) | **0.47 (0.008)** | 0.28 (0.126) | -0.3 (0.096) | **-0.41 (0.024)** | 0.26 (0.160) | 0.35 (0.053) |
| PR (%) | **0.53 (< 0.001)** | | -0.13 (0.461) | 0.01 (0.972) | -0.09 (0.591) | -0.1 (0.576) | 0.07 (0.695) | -0.21 (0.230) | **0.36 (0.030)** |
|  |  |  | |  |  |  |  |  |  |
| Patients who did not undergo PVR between the two measurements in time (n=41) | | | | | | | | | |
| RV EDV (ml/m^2^) | n.s. |  | | **-0.55 (<0.001)** |  |  |  | **-0.35 (0.03)** |  |
| RV ESV (ml/m^2^) | n.s. |  | |  |  | **0.29 (0.049)** |  |  |  |
| RV EF (%) |  |  | |  |  |  |  |  |  |
| RV SV (ml/m^2^) |  |  | |  |  |  |  | n.s. |  |
| RV mass (g) |  | n.s. | |  |  |  |  |  |  |
| RV mass / EDV ratio (g/ml/m^2^) | **-0.45 (0.03)** |  | | **0.54 (0.007)** |  |  |  |  |  |
| PR (%) | **0.54 (0.003)** |  | |  |  |  | 0.**77 (<0.001)** |  | **0.41 (0.03)** |

EDV: end-diastolic volume

EF: ejection fraction

ESV: end-systolic volume

PR: pulmonary regurgitation

RV: right ventricular

SV: stroke volume

PVR: pulmonary valve replacement

| Supplementary table 4: OLINK biomarker measurements complete group | | | | | |
| --- | --- | --- | --- | --- | --- |
|  | **Visit 1** | | **Visit 2** | | **P value** |
|  | Mean | SD | Mean | SD |  |
| NT-proBNP | 4.3 | 1.2 | 3.9 | 1.3 | 0.12 |
| ST2 | 4.6 | 0.6 | 4.7 | 0.7 | 0.54 |
| DLK-1 | 6.7 | 0.8 | 6.8 | 0.9 | 0.58 |
| GDF-15 | 5.2 | 0.6 | 5.3 | 0.6 | 0.46 |
| IGFBP-1 | 5.0 | 1.2 | 4.5 | 1.3 | 0.04 |
| IGFBP-7 | 8.0 | 0.4 | 7.9 | 0.5 | 0.22 |
| VWF | 6.9 | 0.9 | 7.5 | 1.1 | 0.03 |
| FABP-4 | 5.8 | 0.7 | 5.7 | 0.7 | 0.28 |

| Supplementary table 5: changes in OLINK biomarker measurements complete group | | |
| --- | --- | --- |
|  | **Mean** | **SD** |
| NT-proBNP | -0,082 | 0,238 |
| ST2 | 0,009 | 0,081 |
| DLK-1 | 0,008 | 0,080 |
| GDF-15 | 0,002 | 0,085 |
| IGFBP-1 | 0,015 | 0,064 |
| IGFBP-7 | -0,107 | 0,322 |
| VWF | -0,030 | 0,085 |
| FABP-4 | 0,114 | 0,328 |

**Supplementary table 6: correlations between changes in blood biomarkers and left sided CMR measurements (n = 50)**

|  | NT-proBNP | ST2 | GDF-15 | DLK-1 | IGFBP1 | IGFBP7 | vWF | FABP-4 |
| --- | --- | --- | --- | --- | --- | --- | --- | --- |
| LV EDV (ml/m2) | -0.11 (0.454) | 0 (0.989) | -0.16 (0.298) | -0.1 (0.500) | 0.16 (0.290) | -0.08 (0.619) | -0.09 (0.551) | **-0.36 (0.014)** |
| LV ESV (ml/m2) | -0.16 (0.276) | 0 (0.983) | -0.13 (0.402) | -0.1 (0.509) | 0.2 (0.190) | -0.02 (0.901) | -0.08 (0.577) | -0.26 (0.079) |
| LV EF (%) | -0.04 (0.804) | -0.01 (0.933) | -0.13 (0.383) | -0.07 (0.626) | 0.06 (0.714) | -0.1 (0.503) | -0.06 (0.710) | **-0.32 (0.032)** |
| LV SV (ml/m2) | 0.07 (0.646) | 0.04 (0.783) | 0.04 (0.811) | 0.09 (0.544) | -0.13 (0.400) | -0.07 (0.658) | 0 (0.995) | 0.04 (0.801) |
| LV mass (g) | **-0.46 (0.010)** | 0.31 (0.086) | 0.15 (0.411) | 0.06 (0.738) | -0.14 (0.463) | -0.18 (0.326) | -0.12 (0.513) | 0.05 (0.785) |
| LV mass / EDV ratio (g/ml/m2) | **-0.52 (0.003)** | **0.48 (0.006)** | **0.49 (0.005)** | 0.17 (0.347) | -0.29 (0.110) | -0.2 (0.273) | 0 (0.983) | **0.41 (0.021)** |
